# Post-COVID Mucormycosis of Mandible: A Conundrum of Management

**DOI:** 10.7759/cureus.27382

**Published:** 2022-07-28

**Authors:** Ashish Gopal, Raman Sharma, Sakshi Negi, Ishwar Singh

**Affiliations:** 1 Otolaryngology - Head and Neck Surgery, Maulana Azad Medical College, New Delhi, IND

**Keywords:** mandibulectomy, mandible, covid 19, fungal, mucormycosis

## Abstract

Mucormycosis is an acute invasive infection of paranasal sinuses, with the propensity to spread from paranasal sinuses to intra-orbital and cranium. It is usually associated with immunocompromised states like diabetes mellitus, hematological malignancies, long-term steroids, neutropenia, and other multiple systemic disorders. Mucormycosis cases had been upsurging following COVID-19 infection. Due to the rampant use of steroids, pulmonary involvement secondary to cytokine upsurge, and deranged blood sugar levels in diabetic patients, mucormycosis of paranasal sinuses and neighboring anatomical structures has occurred at an unprecedented rate. The isolated involvement of the mandible is infrequent, and very few cases have been reported in the literature. We report one such case of post-COVID-19 isolated mandibular mucormycosis and its management. Generally, surgical excision and reconstruction of defect followed by a course of broad-spectrum antifungals are described as the appropriate line of management for this condition. However, on the contrary, we followed conservative management as the sole treatment because of the various challenges concerning undesirable postoperative consequences and reducing morbidity for the patient.

## Introduction

Mucormycosis (zygomycosis/phycomycosis), first described by Paultauf et al. [1], is a life-threatening infection commonly seen in patients with immunocompromised states like uncontrolled diabetes, hematological malignancies, and long term steroid therapy, neutropenia. It has diverse presentations, including rhino-orbit-cerebral, central nervous system (CNS), pulmonary, gastrointestinal tract (GIT), cutaneous, and vascular manifestations [2]. Amongst these varied presentations, an isolated mucormycosis of the mandible is a rare presentation. Few cases have been reported in the literature, most of which were after dental extraction [3]. Our institute was a dedicated mucormycosis tertiary care center in New Delhi, India, and we managed around 350 patients with debridement and amphotericin. However, we encountered only one case of isolated mandibular mucormycosis.

The risk factors, diagnosis, pathophysiologic features, and treatment are discussed. Various differentials of mucormycosis in this area include bacterial necrotizing infections, carcinoma, aspergillosis, and other fungal diseases, rhinoscleroma, syphilis, tuberculosis, and other granulomatous syndromes [4,5]. In COVID-19 infection, the interaction of several factors like immune dysregulation, steroid therapy, and exacerbation of pre-existing diabetes may allow the mucoralean fungi to cause this disease [6].

## Case presentation

A 67-year-old man presented with the chief complaints of swelling on the left side of his face for six months. The swelling was insidious in onset and non-progressive. The patient was a known hypertensive and was on antihypertensives for the past 10 years with unsatisfactory blood pressure control. The patient was a chronic smoker and used to consume alcohol daily. There was no history of difficulty in mouth opening or swallowing. The patient had contracted a COVID-19 infection three weeks before the presentation that required hospitalization for oxygen support and intravenous steroids support. The steroids were administered on the tenth day of illness in a tapering dose. The patient was a known diabetic, and following steroid treatment, his blood sugar levels got deranged, and he was kept on oral hypoglycemic agents (OHA). The average blood sugar before steroid therapy was 96 mg/dL, which surged to levels ranging between 300-400 mg/dL after the steroid therapy. After that, the patient had a history of paraesthesia and painful loosening of lower central incisors. The pain was radiating to the left temporal region. It was associated with foul-smelling pus discharge from the tooth sockets along with a non-healing ulcer over the gums. The patient then visited a dentist, where an orthopantomogram (OPG) was advised. The OPG demonstrated bony destruction of the alveolar bone of different parts of the mandible (body, ramus, symphysis, and para-symphysis).

**Figure 1:**
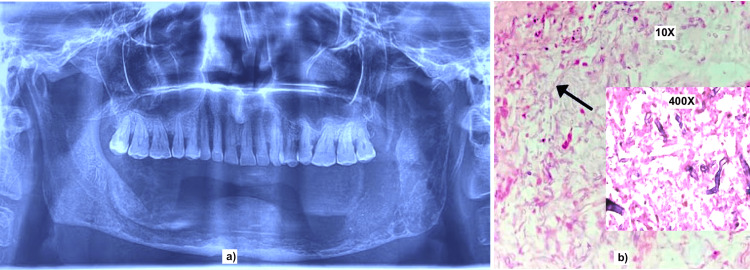
a) Orthopantomogram of the patient showing osseous destruction of the mandible (Body/ ramus/symphysis and parasymphysis). b) Histopathology microphotograph showing broad aseptate hyphae with right-angle branching Eosin stain with 10X and 400X magnification.

Additionally, there were labial and lingual cortical plate erosions throughout (Figure 1a). The patient was then admitted and planned for bilateral inferior marginal alveolectomy by an oromaxillofacial surgeon. Intraoperative findings of dead necrotic cancellous bone with the necrosis of bilateral mental nerves were noted. Curettage-saucerization of the involved mandible segment and complete extraction of all lower teeth were performed. Post-operatively, the decalcification report of the collected pathological tissue demonstrated numerous fungal broad aseptate hyphae with right-angle branching suggestive of Mucormycosis (Figure 1b). After surgery, the patient was on hyperbaric oxygen therapy for 15 days, and Posaconazole was prescribed for the next five months.

**Figure 2:**
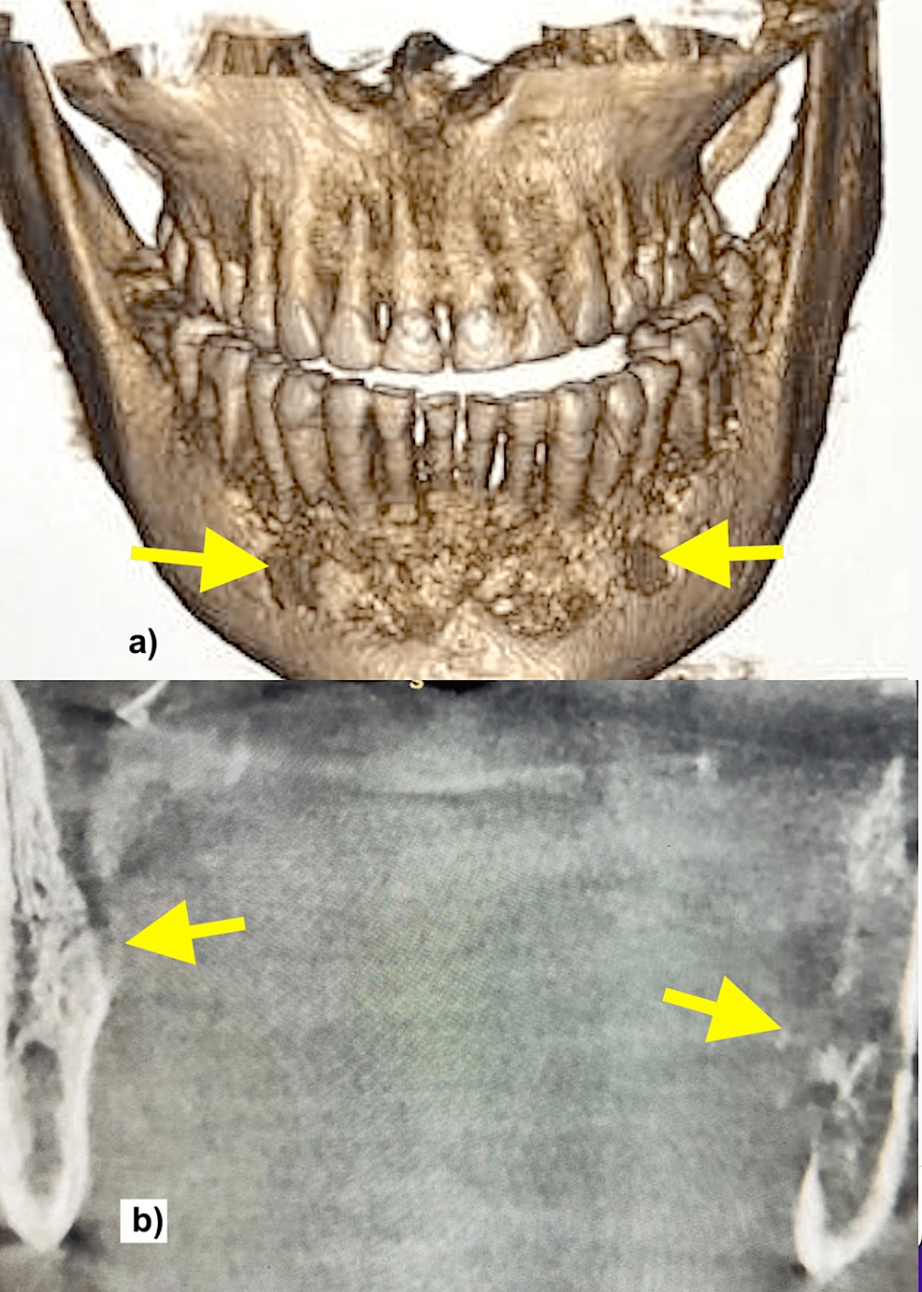
Computed tomography scans of the mandible, three-dimensional reconstruction (a), and coronal cuts (b), showing multiple erosions (arrows).

However, the patient's left parotid swelling was not resolving. Therefore, a cone-beam computed tomography (CBCT) was performed, which showed lytic destruction of the body of both hemimandibles and condylar and coronoid processes of the left side with sequestrum formation (Figure 2). The left masticator muscle appeared bulky and heterogeneous.

**Figure 3:**
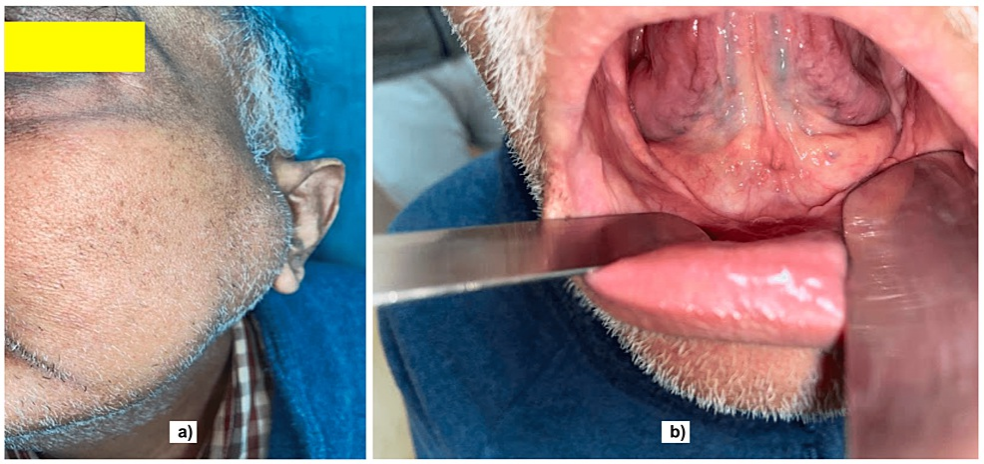
a) Image showing left parotid region swelling. b) Intra-oral image showing the well-healed suture line after marginal alveolectomy.

In addition, mottled air lucency was noted in the body and ramus of the left hemimandible. The patient was referred to our center for initiating amphotericin and further management. On external examination, diffuse swelling in the left parotid region was observed, 4cm x 4cm in dimensions, hard in consistency, and with normal overlying skin (Figure 3a). Intraoral examination showed postoperative changes and a well-healed suture line with no obvious lesion or ulcer (Figure 3b). The patient was then admitted and administered injectable liposomal amphotericin at a loading dose of 1mg/kg 24 hourly on the first day, followed by the maintenance dose of 3mg/kg 24 hourly till the cumulative dose of 10 grams was achieved. The swelling was slightly reduced with the course of antibiotics but not entirely resolved. The patient was not a known diabetic, and OHAs controlled the transient increase in blood sugars post steroid therapy. The OHAs were later stopped as the blood sugars returned to normal, and the patient's HbA1c level was 5.9%. A second CBCT was performed after two months to reassess the progression, which showed involvement of body and ramus of the opposite as well. The surgical plan would have been total mandibulectomy followed by total mandibular titanium prosthesis placement. Still, after discussion with the oromaxillofacial surgeons and explaining the prognosis to the patient about intraoperative and postoperative consequences, the decision was taken not to go for surgical management. Also, the patient refused consent for the surgery when the risks and benefits associated with the surgery were explained. While the total mandibulectomy has the advantages of disease clearance, cosmetic benefit, and functional prosthetic appliance, it carries a risk of immediate airway collapse, tracheostomy dependence, loosening, or extrusion of the prosthesis age and comorbidity-related mortality, recurrence of disease, and high cost. The patient is still on regular follow-up after one year of diagnosis, with no new symptoms or complications. On repeat imaging recently, there were no signs of progression of the disease. Prophylactically, the patient is kept on tablet Posaconazole 300mg once daily and is followed monthly.

## Discussion

Post COVID-19 Mucormycosis is a rapidly progressive disease having a fulminant course and fatal outcomes. A recent study from a tertiary care center in India showed an increased risk of rhino-orbital mucormycosis in patients with uncontrolled diabetes, a dysfunctional immune system due to COVID 19 infection, and injudicious corticosteroids use [7]. Covid 19 infection affects the incompetent innate immune system of the patients [8-10]. In COVID-19 infection, a raised immature neutrophil count cannot eliminate fungal spores allowing them to settle down and germinate into hyphae. There is an upsurge of pro-inflammatory (IL-1, IL-2, IL-6, TNF-Alpha) and anti-inflammatory (IL-4,10) cytokine levels, less CD4 IFN-Gamma expression, and fewer CD4 and CD8 cells, which increase the risk of Invasive fungal infection (mucormycosis) [7]. Steroid therapy can tilt blood glucose levels to hyperglycemia even in healthy individuals and cause steroid-induced diabetes. Steroid therapy and Diabetes coexistence can augment immunosuppression and hyperglycemia, increasing the risk of infection [11]. Hyperglycaemia, acidosis, and injudicious corticosteroid use alter the phagocytic functions of phagocytes, impair chemotaxis and defective intracellular killing, the principal host defense mechanism against mucormycosis, to immigrate to infected tissue and kill the organism [12].

Both clinical and radiological assessment of the disease is of utmost importance. At times radiology does not give an accurate picture of the disease that we explore during surgery, which is much more extensive than preoperative scans [13]. After diagnosing the disease, Amphotericin-B and early aggressive surgical debridement are the treatment of choice [14]. The most frequently involved site in the oral and maxillofacial region is the maxillary sinus, where it presents with tissue necrosis and can extend to orbit and the brain [15]. However, mandibular mucormycosis is extremely rare. The first case of mandibular mucormycosis was reported by Eisenberg et al. [2]. The mainstay of treatment of mucormycosis is early diagnosis, removal of risk factors, debridement of infected tissue, surgical resection, and effective antifungal therapy [16]. In our case, initially, a marginal alveolectomy was done, and the patient was given oral Posaconazole. The disease successively involved the remaining mandible.

We formulated our initial plan to remove and debride the mandible and reconstruct the mandible using a titanium prosthesis at a second stage. We figured that the tissue planes would have adhered and no longer existed, thus making a surgical challenge. Three cases of isolated mandibular mucormycosis have been reported in the study from the All India Institute of Medical Sciences, Bhopal (IN), where segmental debridement was done, followed by reconstruction using titanium implants [13], unlike in our case of total mandible bone involvement. The postoperative follow-up and assessment of the quality of life are of utmost importance, which was not mentioned in any of the cases. Total mandibulectomy with reconstruction and postoperative management is of great importance, so we explained to the patient the possible options for every part of the surgery. In our case, we explained to the patient various reconstruction options and postoperative results; after detailed discussion and counseling, the patient and attendants opted out of the surgical option.

While the total mandibulectomy has the advantages of disease clearance, cosmetic benefits, and functional prosthetic appliance, the disadvantages include: Immediate airway collapse after total mandible removal with glossoptosis, and the patient will be tracheostomy dependent; The inflamed tissues around the necrosed mandible may not be suitable to hold the newly placed implant at the time of first surgery; Second-stage surgery faced with the dilemma of adhered or lost tissue planes; The risk of implant extrusion in later stages because of old age will always be present; The patient may not be able to tolerate the surgical procedure at this age; hence surgical intervention will increase morbidity and mortality; The patient will not have normal mastication, and there are more chances of drooling due to inadvertent injury to bilateral marginal nerves [17]; The patient has to undergo one more procedure, i.e., tracheostomy hence will lose his voice, and other disadvantages associated with tracheostomy will add up, further increasing the morbidity; The risk of recurrence of disease; Total titanium mandibular prosthesis may not be economically feasible for all.

After keeping all the above points in mind, it was decided to keep the patient over medical management as total mandibulectomy will further deteriorate the patient's physical, mental and social life and has more risk than benefit for the patient's health. Hence mandible was not removed, and the patient was discharged over tablet Posaconazole and is kept on follow-up.

## Conclusions

Isolated mandible involvement after COVID-19 is a rare presentation of mucormycosis. Surgical debridement followed by the course of amphotericin is the recommended treatment. However, it should be attempted with utmost care in a case of total mandibular involvement to circumvent the intraoperative or postoperative consequences. A good clinical course can be expected without requiring total mandibulectomy and prosthesis replacement with medical management and close follow-up.

## References

[1] Paltauf A (1885). Mycosis mucorina: Ein Beitrag zur Kenntnis der menschilchen Fadenpiltzer-krankungen [German]. Virchows Arch Pathol Anat.

[2] Eisenberg L, Wood T, Boles R (1977). Mucormycosis. Laryngoscope.

[3] Gupta N, Kumar R, Soneja M, Singh G, Khot W, Malla S, Xess I (2019). Mucor menace in an immunocompetent young male after dental manipulation. J Family Med Prim Care.

[4] Koopmann CF Jr, Coulthard SW (1982). Infectious facial and nasal cutaneous necrosis: evaluation and diagnosis. Laryngoscope.

[5] Klabacha ME, Stankiewicz JA, Clift SE (1982). Severe soft tissue infection of the face and neck: a classification. Laryngoscope.

[6] Moorthy A, Gaikwad R, Krishna S (2021). SARS-CoV-2, uncontrolled diabetes and corticosteroids-an unholy trinity in invasive fungal infections of the maxillofacial region? a retrospective, multi-centric analysis. J Maxillofac Oral Surg.

[7] Meher R, Wadhwa V, Kumar V (2022). COVID associated mucormycosis: a preliminary study from a dedicated COVID hospital in Delhi. Am J Otolaryngol.

[8] Gupta A, Sharma A, Chakrabarti A (2021). The emergence of post-COVID-19 mucormycosis in India: can we prevent it?. Indian J Ophthalmol.

[9] Schulte-Schrepping J, Reusch N, Paclik D (2020). Severe COVID-19 Is marked by a dysregulated myeloid cell compartment. Cell.

[10] Parackova Z, Zentsova I, Bloomfield M (2020). Disharmonic inflammatory signatures in COVID-19: augmented neutrophils' but impaired monocytes' and dendritic cells' responsiveness. Cells.

[11] Ardi P, Daie-Ghazvini R, Hashemi SJ (2020). Study on invasive aspergillosis using galactomannan enzyme immunoassay and determining antifungal drug susceptibility among hospitalized patients with hematologic malignancies or candidates for organ transplantation. Microb Pathog.

[12] Corzo-León DE, Chora-Hernández LD, Rodríguez-Zulueta AP, Walsh TJ (2018). Diabetes mellitus as the major risk factor for mucormycosis in Mexico: epidemiology, diagnosis, and outcomes of reported cases. Med Mycol.

[13] Dasukil S, Boyina KK, Sarkar S (2021). Management of Covid associated mucormycosis of mandible: a mountain beneath a molehill-a lesson learnt. Indian J Otolaryngol Head Neck Surg.

[14] Brown OE, Finn R (1986). Mucormycosis of the mandible. J Oral Maxillofac Surg.

[15] Kwak EJ, Kim DJ, Nam W, Park W (2020). Mucormycosis in the jaw: a report of 2 cases and literature review. Oral Health Prev Dent.

[16] Kontoyiannis DP, Lewis RE (2011). How I treat mucormycosis. Blood.

[17] Warshavsky A, Fliss DM, Frenkel G (2019). Quality of life after mandibulectomy: the impact of the resected subsite. Int J Oral Maxillofac Surg.

